# Editorial: Preserving antibiotics: stewardship and effective treatment in low and middle income countries

**DOI:** 10.3389/frabi.2024.1432477

**Published:** 2024-05-30

**Authors:** Abdul Ghafur, Stephen H. Gillespie

**Affiliations:** ^1^ Apollo Cancer Institute, Apollo Speciality Hospitals, Chennai, India; ^2^ Division of Infection and Global Health, School of Medicine, University of St Andrews, St Andrews, United Kingdom

**Keywords:** AMR, infection control (IC), antibiotic stewardship (ABS), health policy, microbiome

In an era where antimicrobial resistance (AMR) threatens global health, understanding and addressing its escalation, particularly in low and middle-income countries (LMICs), becomes crucial. It is clear that this complex problem can only be solved by a wide-ranging multidisciplinary approach. This editorial synthesizes findings from six pivotal articles, that provide valuable insights into different aspects of AMR, from stewardship and public awareness to the implications of malnutrition on antimicrobial effectiveness.

It is often said that information is power and we hope that informing patients will have an impact on decision making. Thus, public reporting of prescription indicators has been considered to be a useful tool to modulate antibiotic prescribing and usage behaviours. (Chen M et al.). According to the first article, while public awareness of such reporting correlates with a better understanding of prescription practices, it does little to alter patient choices. This finding implies that simply providing information is insufficient to change behaviours and highlights the need for more interactive and comprehensive educational approaches that actively engage both healthcare providers and patients in understanding the importance of prudent antibiotic use.

Controlling the rise in AMR is especially challenging in Low and Middle Income Countries (LMIC) as exemplified by the experiences of Queen Elizabeth Central Hospital in Malawi reported in the second article. This group implemented a series of AMR control activities, including rigorous antimicrobial stewardship programs and enhanced laboratory diagnostics, to combat the rise of drug-resistant infections. These efforts are foundational, not only did their efforts reduce the incidence of AMR but also showed what is possible for other hospitals in similar resource-limited settings. The success of these initiatives relies heavily on local leadership and tailored educational programs for healthcare workers, emphasizing the critical role of context-specific strategies (Kamalo et al.).

Point-of-care testing (POCT) for C-reactive protein (CRP) is another innovative approach to reducing unnecessary antibiotic prescriptions, as explored in the third article. By enabling the distinction between bacterial and viral infections, CRP POCT supports more judicious use of antibiotics. This tool is especially valuable in primary care, particularly in settings where over prescription is driven by diagnostic uncertainty (Van Hecke et al.).

The fourth article delves into the broader challenges of antibiotic use and prescribing practices, identifying a pervasive issue of overuse across various healthcare settings. The article calls for stronger antimicrobial stewardship measures to curb this trend, suggesting that more stringent guidelines and training programs are essential to ensure that antibiotics are prescribed only when absolutely necessary, thereby preserving their efficacy for future generations (Kamita et al.).

The nutritional status of patients plays a crucial role in the effectiveness of antimicrobial treatments, as detailed in the fifth article. In malnourished children, disrupted gut microbiomes contribute to heightened susceptibility to infections that are harder to treat due to AMR. This relationship underscores the importance of a holistic approach to AMR, addressing nutritional deficiencies and other concomitant health problems as part of a comprehensive strategy. This is especially important in regions where malnutrition is prevalent (Arredondo-Hernandez et al.).

Finally, the sixth article advocates for a unified global approach to tackle AMR, emphasizing the interconnectedness of human, animal, and environmental health. The One Health approach it proposes is essential, recognizing that AMR is not just a medical issue but a complex ecological problem that requires coordinated international efforts (Veerapa-Mangroo et al.).

This Research Topic confirms that the challenge of AMR is daunting, particularly in LMICs. Yet using the tools described here there is hope that the combined insights from these articles provide can make a difference. They show that through informed policies, collaborative efforts, and innovative practices tailored to local needs, it is possible to preserve the effectiveness of antibiotics. As the global community continues to grapple with AMR, these findings serve as a crucial guide for future action, highlighting the importance of integrated approaches that consider the environmental, nutritional, and socio-economic factors at play. The journey to overcoming AMR is long and complex, but with sustained commitment and global cooperation, progress is within reach.

## Author contributions

AG: Writing – review & editing, Writing – original draft. SG: Writing – review & editing, Supervision, Conceptualization.

